# Enhanced Silver Nanoparticle Synthesis by *Escherichia Coli* Transformed with *Candida Albicans* Metallothionein Gene

**DOI:** 10.3390/ma12244180

**Published:** 2019-12-12

**Authors:** Qunying Yuan, Manjula Bomma, Zhigang Xiao

**Affiliations:** 1 Department of Biological and Environmental Science, Alabama A&M University, Normal, AL 35762, USA; mbomma@bulldogs.aamu.edu; 2 Department of Electrical Engineering and Computer Science, Alabama A&M University, Normal, AL 35762, USA; zhigang.xiao@aamu.edu

**Keywords:** biosynthesis, silver nanoparticles, engineered *Escherichia coli*, metallothionein

## Abstract

In this study, the metallothionein gene of *Candida albicans* (*C. albicans*) was assembled by polymerase chain reaction (PCR), inserted into pUC19 vector, and further transformed into *Escherichia coli (E. coli*) DH5α cells. The capacity of these recombinant *E. coli* DH5α cells to synthesize silver nanoparticles was examined. Our results demonstrated that the expression of *C. albicans* metallothionein in *E. coli* promoted the bacterial tolerance to metal ions and increased yield of silver nanoparticle synthesis. The compositional and morphological analysis of the silver nanoparticles revealed that silver nanoparticles synthesized by the engineered *E. coli* cells are around 20 nm in size, and spherical in shape. Importantly, the silver nanoparticles produced by the engineered cells were more homogeneous in shape and size than those produced by bacteria lack of the *C. albicans* metallothionein. Our study provided preliminary information for further development of the engineered *E. coli* as a platform for large-scale production of uniform nanoparticles for various applications in nanotechnology.

## 1. Introduction

Nanoparticles (NPs) have been explored for a wide range of applications, including biosensors and chemical sensors, bioimaging, catalysis, optics, electronics, drug delivery, and energy [1]. The conventional chemical or physical methods for the production of nanoscale materials depend on the utilization of toxic stabilizing and capping agents, which pose risks to the environment and human health [2]. Therefore, there is an increasing need to develop cost-effective, non-toxic, and environmentally green approaches that do not use toxic chemicals to produce NPs. The microbial synthesis of NPs, which takes advantage of microbial detoxification of metallic ions and precipitation of metals through reducing enzymes, intracellular or extracellular reducing reagents and metal-binding proteins, showed immense potential [3,4].

In particular, there is an increasing interest in the exploitation of the cysteine-rich metal-binding proteins, metallothioneins (MTs), in the development of biofactories for metal NP production. MTs are found in all eukaryotes, as well as some prokaryotes, and play an important role in intracellular metal distribution, accumulation, as well as protection of cells against heavy metal toxicity and oxidative stress [5,6]. The Cys-Xaa-Cys clusters of MTs can act as a reducing functional group for the reduction of metal ions, chelation, and accumulation of metal particles in the cells [5,6,7]. Studies showed that MTs are involved in the detoxification of several metals, including zinc, mercury, copper and cadmium in microorganisms [5,6,8]. Due to their important role in metal reduction and high metal binding affinity, studies have been seeking to integrate MTs in creating biological platforms for the production of metal NPs. Engineered bacteria expressing MT and/or phytochelatins have been shown to be able to produce diverse nanoparticles [9,10,11].

A critical aspect in developing a microbial platform for NP synthesis is choosing a proper host microorganism. It is ideal that a biofactory can make NPs with consistent properties for their applications in various field of nanotechnology. Many types of microorganism have been shown to be able to synthesize various metal NPs, but with diverse properties [12,13,14,15,16]. In addition, the conditions for cultivation and maintenance of microorganisms vary for different species [14,15,16]. Among the microorganisms able to serve as potential biofactories for metal NP synthesis, the readily available bacteria, *E. coli*, is of particular interest, due to the relatively easy and simple procedures to grow, maintain and manipulate them in the lab. The potential of engineered *E. coli* as a common platform for synthesis of diverse NP has been explored; however, the efficiency of NP synthesis by the engineered bacteria and the potential to upgrade to large scale NP production were not examined [9,10,11].

The goal of this study was to provide preliminary evidence for exploiting engineered *E. coil* as a platform for the large-scale production of metal NPs with controlled morphological properties. In this study, we chose *C. albicans* MT as a target protein and transferred a PCR-assembled *C. albicans* MT gene into *E. coli* DH5α cells to examine whether expression of *C. albicans* MT in *E. coli* would improve bacterial NP synthesis. 

Compared to *E. coli* MT, which has four cysteine out of 56 residues (7.1%), the *C. albicans* MT has a relatively high content of cysteine residues (15.8%). Importantly, the *C. albicans* has four repeats of Cys-Xaa-Cys presumably for binding metal ions, such as copper [17,18]. To examine whether the expression of this MT protein will improve NP synthesis, we used biosynthesis of silver NPs as an example to investigate the effects of *C. albicans* MT on NP production. Our results showed that the *E. coli* cells expressing *C. albicans* MT produced silver NPs faster than *E. coli* cells transformed with pUC19 empty vectors. Particularly, the silver NPs synthesized by engineered *E. coli* are more uniform in size and shape.

## 2. Materials and Methods

### 2.1. Synthesis of C. Albicans MT Gene and Transformation of E. Coli DH5α Cells

To obtain the *C. albicans* MT gene, first, the MT protein sequence of the *C. albicans* was obtained from the NCBI protein database. Then the *C. albicans* MT gene was codon-optimized for protein expression in *E. coli*, and eight oligonucleotides for gene synthesis were designed using DNAworks. The oligonucleotides were synthesized by Eurofins Genomics (Louisville, KY, USA). The *C. albicans* gene was synthesized in four consecutive PCR reactions, as described by D. Marsc [19]. Assembled gene was inserted between HindIII and BamHI sites of plasmid pUC19 through in vivo homologous recombination in *E. coli* DH5α cells. Plasmids were propagated in *E. coli* DH5α cells, and clones carrying the error-free gene are confirmed by DNA sequencing (Eurofins Genomics, Louisville, KY, USA). To obtain genetically modified *E. coli* cells for NP synthesis, a single colony of *E. coli* DH5α cells transformed with the sequence-verified plasmid was inoculated in 5 mL of Luria-Bertani (LB) medium in the presence of 100 µg/mL of carbenicillin and cultured overnight at 37 °C with a continuous shake at 250 rpm. The overnight culture was centrifuged using Sorvall Legend X1R (Fisher Scientific, Pittsburgh, PA, USA) and the cell pellet was resuspended in 15 mL of fresh LB medium containing 100 µg/mL of carbenicillin. The cells were further cultured at 37 °C with a continuous shake for 30–45 min. Then glycerol was added to the cells to reach a final concentration of 15% and cells were stored at −80 °C for future use. All chemicals and reagents were purchased from Fisher Scientific (Pittsburgh, PA, USA) unless specified.

### 2.2. Monitoring Cell Growth

To examine whether the expression of *C. albicans* MT in E. coli affects cell growth, we monitored cell growth along time of cultivation. 50 µL of the glycerol stock cells containing the *C. albicans* MT gene inserted in pUC19 plasmid (pUC-Met) was inoculated in 10 mL of LB in the presence of 100 µg/mL carbenicillin and cultured overnight at 37 °C in a shaker incubator. Cells were spun down and resuspended in 100 mL of LB with 100 µg/mL carbenicillin. Optical density at 600 nm (OD_600nm_) was monitored each hour after resuspension until OD_600nm_ reached between 0.6 and 0.8, then OD_600nm_ was measured at 24 h, 48 h and 72 h. It has been shown that MT expression was induced and upregulated when microorganisms are under heavy metal stress [5,6,17,18]. To further investigate whether the expression of *C. albicans* MT in *E. coli* impact the bacterial tolerance to heavy metal, we cultivated the bacteria in the presence of 0.2 mM CuSO_4_. 0.2 mM of CuSO_4_ was added to the cells after OD_600nm_ reached between 0.6 and 0.8. Cells were further cultivated at 37 °C for 17–19 h with a continuous shake. OD_600nm_ was measured again before cells were collected by centrifugation at 4000× *g* for 10 min. *E. coli* cells transformed with pUC19 empty vector (pUC19-Vec) was used as control.

The viability of the cells after they grew overnight in the presence of 0.2 mM CuSO_4_ was estimated by plating the cells from the overnight culture on agar plates. The overnight bacterial culture was diluted with LB medium at a ratio of 1:1000, 1:10,000, 1:100,000 and 1:1,000,000, and 100 µL of each dilution was plated on agar plates in the absence of CuSO_4_. Cells were allowed to grow overnight at 37 °C for 17 h to obtain colonies. Individual colonies were counted to estimate the viable cells after CuSO_4_ challenge. 

### 2.3. Determination of MT Protein Expression

*C. albicans* MT was identified as a copper binding protein, and its level increases under metal ion stresses [5,6,17,18]. To analyze the expression level of *C. albicans* MT in *E. coli*, the preparation of overnight culture for examining protein expression was carried out similarly as described in “Monitoring Cell Growth”. The overnight cell culture was centrifuged, and cells were resuspended in 100 mL of LB containing carbenicillin and grown at 37 °C. When OD_600nm_ reached between 0.6 and 0.8, bacteria were grown for another 8 h for MT protein expression analysis. Cell pellets were resuspended in lysis buffer containing 50 mM Tris-HCl, pH 8, 50 mM NaCl at a ratio of about 0.05 g wet cells/mL of buffer and sonicated to break apart the cells. The whole lysate was centrifuge again at 15,000× *g* for 30 min to separate the soluble proteins from the cell membrane debris. Soluble proteins were passed through a GE Healthcare Vivaspin™ concentrator with a 30-kDa cutoff to remove the proteins larger than 30 kDa. Subsequently, the flow through was further concentrated with GE Healthcare Vivaspin™ concentrator with a 3-kDa cutoff. 10, 20, 40 µg of the concentrated proteins smaller than 30 kDa were run on 16% T, 4% C Tricine SDS-PAGE gel to separate the proteins. Proteins were stained in buffer containing 30% methanol, 10% glacial acetic acid, 0.1% Coomassie blue and 60% water for at least 2 h. Then the gel was destained in the same buffer without Coomassie blue, for overnight with continuous shake. Gel picture was taken using Gel Doc EZ Gel Documentation System (Biorad, Hercules, CA, USA).

### 2.4. Biosynthesis of Silver NPs by Bacteria

The preparation of overnight culture for silver NP synthesis was carried out similarly as described in “Monitoring Cell Growth”. The overnight cell culture was centrifuged, and cells were resuspended in 100 mL of LB in the presence of carbenicillin and grown at 37 °C. AgNO_3_ was added to the culture when OD_600nm_ reached between 0.6–0.8. Cells were further cultured at 37 °C with continuous shaking for 72 h. Samples were taken to measure OD_600nm_ at 24 h, 48 h and 72 h after addition of AgNO_3_. To estimate the yield of silver NPs in a larger scale, we carried out three experiments in which bacteria were grown in 1 L LB medium in the presence of 1 mM AgNO_3_ under the same conditions as for small scales. The wet weight of pellet collected after 72 h of cultivation was measured. After each OD_600nm_ measurement, cultures sat for 30 min to allow particles to settle down on the bottom of the flask and take pictures of the precipitates. pUC19-Vec cells were used as a control in this experiment.

### 2.5. Collection of NPs

After 72 h of cultivation, cell culture was centrifuged at 4000× *g* for 10 min. The cell pellets were resuspended in 1.5 mL of 4 M urea, sonicated for three cycles of 45 s at an output of 20%, with an interval of 1 min between each cycle (Sonic Dismembrator, Model 50; Fisher Scientific, Pittsburgh, PA, USA). The lysate was then transferred to a 2-mL microcentrifuge tube and further spun at 10,000× *g* for 10 min (accuSpin™ Microcentrifuge, Fisher Scientific, Pittsburgh, PA, USA). The supernatant was discarded, and the pellet was resuspended in 1 mL of 4 M urea by 3–4 pulses of sonication, followed by centrifugation to remove the supernatant. The pellet was further washed twice with 1 mL of sterile deionized water. The final pellet was resuspended in sterile deionized water and stored in the dark at 4 °C for next step characterizations. 

### 2.6. Fourier Transform Infrared (FTIR) Spectral Analysis

FTIR was used to reveal the functional groups that may be responsible for reducing and stabilizing the bioreduced silver NPs. The isolated NPs were diluted in deionized water, and The FTIR spectra were collected at a resolution of 2 cm^−1^ in transmission mode range between 4000 and 500 cm^−1^ using NICOLET IS10 FTIR (ThermoFisher, Waltham, MA, USA). Sterile deionized water was used as blank for this experiment. 

### 2.7. Scanning Electron Microscopic and Scanning Transmission Electron Microscopic Characterization

We used scanning electron microscopy (SEM) and scanning transmission electron microscopy (STEM) to determine the morphological properties of the biologically synthesized silver NPs. The samples for SEM and TEM were prepared similarly. NPs washed with urea and deionized water were resuspended in sterile deionized water. 

An FEI Tecnai F30 super-twin field-emission-gun transmission electron microscope (TEM) (manufactured by the FEI Company in Hillsboro, OR, USA) operating at 300 kV was used to acquire the TEM images, electron diffraction patterns and annular dark-field (ADF) scanning transmission electron microscopy (STEM) images. Chemical composition was explored using an X-ray energy dispersive spectrometer (EDS) attached to the TEM. A dual-beam focus ion beam (FIB) (FEI Helios 600 dual-beam FIB) was used to obtain the SEM images of the biosynthesized silver NPs. The size of NPs was analyzed using Gatan Digital Micrograph Software.

### 2.8. Statistical Analysis

All data are expressed as mean ±SD. Comparison between groups was evaluated with the Student t-test. Probability values of <0.05 were considered significant.

## 3. Results

### 3.1. MT Gene Assembly in vitro and Expression of MT in E. coli DH5α Cells

**Figure 1:**
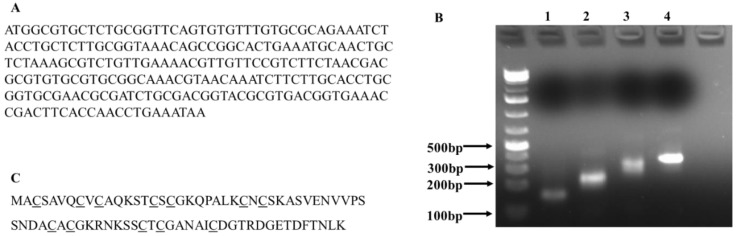
Gene assembly of *Candida albicans* metallothionein gene. (**A**): The nucleotide sequence of *C. albicans* metallothionein (GenBank: KHC50670.1). The percentage of cysteine residues (underscored) in the full peptide is ~15.8%. Its calculated molecular weight is 7.8 Kd; (**B**): PCR-based synthesis of the *C. albicans* metallothionein gene; (**C**): The peptide sequence of *C. albicans* metallothionein.

We synthesize the *C. albicans* MT gene instead of cloning it from the *C. albicans’* genome for two reasons. First, we could obtain the gene without the need of *C. albicans’* genomic DNA; second, it allowed us to optimize codons for improving the expression of a yeast protein in *E. coli*. Eight oligonucleotides covering the full length of *C. albicans* MT gene were designed to synthesize the gene by PCR followed the procedure described in reference 19. The full length of *C. albicans* MT gene was assembled in four consecutive PCR reactions (Figure 1A,B). The expected length of the final PCR product is 288 base pairs the product of each PCR reaction was run on a 1% agarose gel. The incremental increase in DNA size indicated the successful assembly of the gene (Figure 1B). The full-length *C. albicans* MT protein has 76 residues with a calculated molecular weight of 7.8 kDa, and has 15.8% (12/76) cysteine residues (Figure 1C).

**Figure 2:**
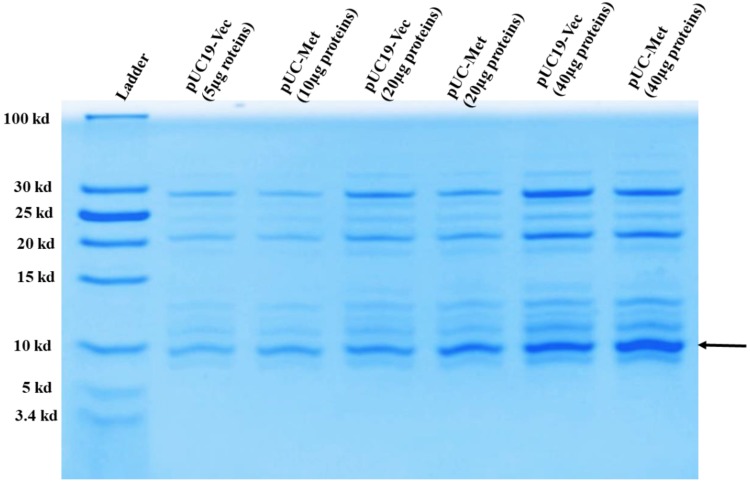
Expression of *C. albicans* metallothionein. 10, 20, 40 µg of soluble proteins smaller than 30 kDa from pUC19-Vec and pUC-Met group were run on 16% T, 4% C Tricine SDS-PAGE gel and stained with Coomassie blue.

16% T, 4% C Tricine SDS-PAGE and Coomassie blue staining were used to analyze the expression of *C. albicans* MT protein in *E. coli*. Figure 2. showed the intensity of a band with a size between 5–10 kDa was enhanced in the proteins smaller than 30 kDa from pUC-Met cells (arrow pointed). The size of this band in pUC-Met cells matched the predicted molecular weight of the *C. albicans* MT protein; while the band with a similar size was much weaker in pUC19-Vec cells. It is possible that the *C. albicans* MT was co-localized with an endogenous protein. In addition, the insertion of the *C. albicans* metallothionein gene between BamHI and HindIII of pUC19 added a few more residues to the original peptide and increased its molecular weight to ~8.5 kDa. We believe that the increase in the intensity of this band was due to the expression of the recombinant *C. albicans* MT.

### 3.2. Cell Growth and Survival during Extended Cultivation and Copper Ion Challenge

**Figure 3:**
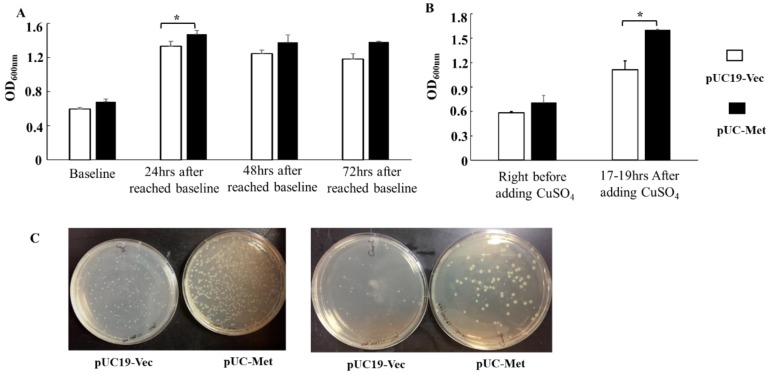
Bacterial cell growth during extended cultivation and upon CuSO_4_ challenge. (**A**): D_600nm_ measurements of bacteria culture at different time points during 72-h cultivation in the absence of metal ions. The baseline measurement was taken when the OD_600nm_ of pUC19-Vec bacterial culture reached around 0.6 (n = 7 for baseline and 24-h groups, *: *p* < 0.05. n = 3 for 48-h and 72-h groups); (**B**): OD_600nm_ measurements of bacteria culture before and 17–19 h after adding 0.2 mM CuSO_4_ (n = 4, *: *p* < 0.05); (**C**): Cell viability after 0.2 mM CuSO_4_ challenge. The cells after 17 h of cultivation in LB containing 0.2 mM CuSO_4_ were plated on agar plates at 1: 100,000 dilution (left panel) and 1:1,000,000 dilution (right panel), and further incubated overnight at 37 °C.

Once we revealed that the *C. albicans* MT protein was expressed in *E. coli*, we continue to explore whether its expression can facilitate bacterial growth, as well as metal NP biosynthesis. Our results supported the notion that the expression of *C. albicans* MT protein promoted bacterial growth and survival. Under the same cultivation conditions, it appeared that the engineered recombinant pUC-Met cells grew faster and survived better than the pUC19-Vec cells during extended cultivation. We chose OD_600nm_ between 0.6 and 0.8 as the baseline for monitoring growth rate, because this OD_600nm_ range usually indicates the bacteria are at their fastest-growing stage and an inducer is added to induce protein expression if the target gene is under the control of an inducible promoter. When the starter culture was similar, at the point when OD_600nm_ of pUC19-Vec cells reached between 0.5 and 0.6 (0.59 ± 0.044, n = 7), the OD_600nm_ of pUC-Met cells was between 0.6 and 0.8 (0.68 ± 0.097, n = 7) (Figure 3A,B). At 24 h after OD_600nm_ reached between 0.6 and 0.8, the OD_600nm_ measurement of engineered pUC-Met cells was significantly higher than that of pUC19-Vec cells (Figure 3A. OD_600nm_: 1.33 ± 0.15 vs. 1.47 ± 0.12; *p* < 0.05, n = 7). Similar trends were observed at 48, 72 h of cultivation, even if the measurements were not significantly different (Figure 3A). Furthermore, the pUC-Met cells performed better than the pUC19-Vec cells when the cells grew 17–19 h in the presence of 0.2 mM CuSO_4_. The growth of pUC-Vec cells was significantly slowed down, compared to the pUC-Met cells (Figure 3B. OD_600nm_: 1.11 ± 0.21 vs. 1.61 ± 0.008, n = 3, *p* < 0.05). These results suggested that the expression of *C. albicans* MT helped cell grow and survive during extended cultivation and under heavy metal challenge. To determine that the higher OD_600nm_ was due to higher survival of pUC-Met cells under stress, we plated the bacteria on agar plates in a serial of dilution after they were exposed to 0.2 mM CuSO_4_ for 17–19 h. Results of 1:1000 and 1:10,000 dilution were not shown because there were too many colonies, and it was not easy to count the individual colonies. Results of 1:100,000 and 1:1,000,000 dilutions were shown in Figure 3C. We found that that the pUC-Met cells after overnight cultivation in medium containing CuSO_4_, not only gave rise to more colonies, but also these colonies were larger than the ones from pUC19-Vec cells (Figure 3C). The estimated viable cell number after overnight CuSO_4_ challenge was 6.4 × 10^8^ in the pUC-Met group, while it was 2.4 × 10^8^ in pUC19-Vec group. Thus, we demonstrated that the expression of *C. albicans* MT increased the tolerance of recombinant *E. coli* to copper ion and facilitated cell survival under heavy metal stress.

### 3.3. Yield of Silver NPs

**Figure 4:**
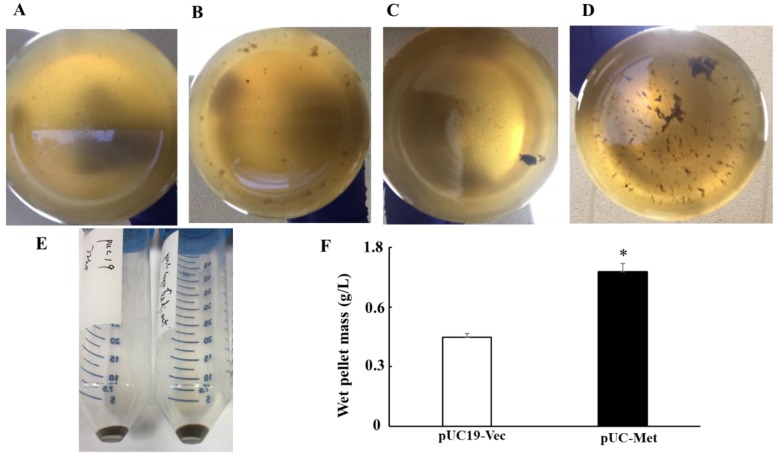
Silver nanoparticle yield of bacteria. Representative pictures were taken after culture sat for 30 min to allow the precipitates to settle down. (**A**,**B**): pUC19-Vec cells (**A**) and pUC-Met cells (**B**) 24 h after addition of 1 mM AgNO_3_; (**C**,**D**): pUC19-Vec cells (**C**) and pUC-Met cells (**D**) 72 h after addition of 1 mM AgNO_3_; (**E**): Pellets collected after 72 h of cultivation in 100 mL medium containing 1 mM AgNO_3_. Left tube: From pUC19-Vec cells; right tube: From pUC-Met cells; (**F**): Quantitation of wet weight of pellets from 1 L bacteria culture in the presence of 1 mM AgNO_3_. *: *p* < 0.05, n = 3.

After we found that expression of *C. albicans* MT helped bacteria grow and improved their resistance to metal ions, we went on to examine whether this would allow the bacteria to produce NPs with improved yield and quality. A color change in the medium after addition of AgNO_3_ to the bacteria culture is usually a sign of NP formation. We not only observed a color change from yellow to brown, but also detected a large amount of black precipitates in all groups of cells. Pictures of precipitates at the bottom of the flasks were taken after culture containers sat at room temperature for 30 min. We observed more dark precipitates formed by pUC-Met cells (Figure 4A–D) at 24 and 72 h after addition of AgNO_3_. Importantly, the wet weight of pellets obtained after centrifugation of 1 L bacterial culture was greater than that of pUC19-Vec cells (0.89 ± 0.04 g/L in pUC19-Vec vs. 1.55 ± 0.084 g/L in pUC-Met. N = 3. *p* < 0.05) (Figure 4F). Thus, our data suggested that the expression of MT facilitated cell survival, as well as the yield of NP production. 

### 3.4. Identification of the Functional Groups Involved in Reducing and Stabilizing Silver NPs

**Figure 5:**
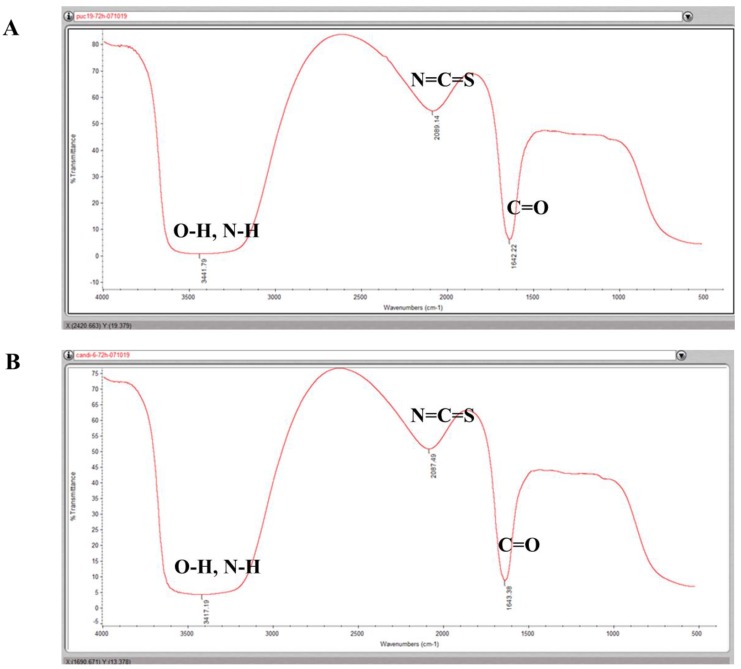
FTIR spectra scanning of silver nanoparticles (NPs). (**A**): FTIR spectra of NPs produced by pUC19-Vec cells; (**B**): FTIR spectra of NPs produced by pUC19-Met cells. In both groups, four potential functional groups were observed: Phenol (O–H), amine (N–H), isothiocyanate (N=C=S) and amide I (C=O).

The mechanisms of bacterial biological synthesis of metal NPs involve the presence of metal-binding protein peptides, reducing enzymes and functional groups that can donate electrons in the bacteria [4,7,12,13,20,21,22]. FTIR is able to identify the chemical composition of biological samples. We used FTIR to analyze the possible reducing functional groups in the bacteria that are involved in reducing and stabilizing silver ions. Three peaks were identified in the silver NP samples around 1640 cm^−1^, 2080 cm^−1^ and 3440 cm^−1^. The sharp, strong peak at 1640 cm^−1^ was probably the stretch of amide I bonds (C=O), from the conversion of C–OH biomolecules. The absorption peak at 1640 cm^−1^ shows that proteins are interacting with biosynthesized nanoparticles, and also their secondary structure were not affected during reaction with Ag^+^ ions or after binding with silver NPs [23,24], while the peak at around 2090 cm^−1^ was likely the stretch of isothiocyanate group (N=C=S). The broad strong stretch of compounds, ranging from 3200 cm^−1^ to 3600 cm^−1^, were assigned to both phenols (O–H) and amines (N–H) (Figure 5). These results were generally similar to what has been reported with the exception of isothiocyanate compounds [23,24]. There were no differences in the peaks between the two groups of cells, suggesting that the bacteria probably used similar functional groups in the reduction of metal ions and the formation of NPs.

### 3.5. Characterization of Silver NPs Using Scanning Electron Microscopy and Scanning Transmission Electron Microscopy

To confirm that bacteria did produce silver NPs, and to determine the compositions and morphological properties of the NPs, we examined the NPs using EDS, SEM and Scanning TEM. 

**Figure 6:**
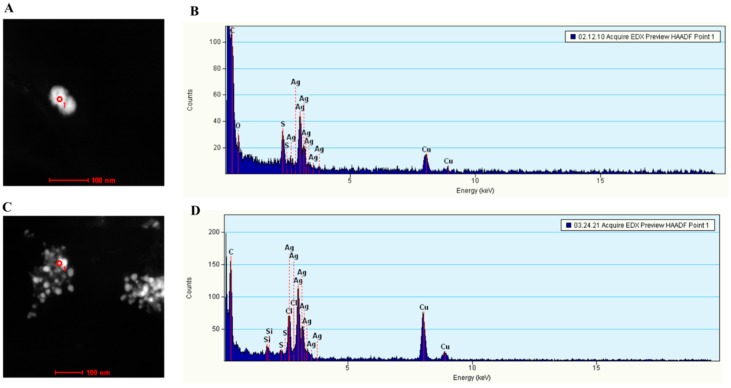
STEM compositional analysis of NPs. (**A**): A representative NP synthesized by pUC19-Vec cells was chosen for compositional analysis; (**B**): Elements in the NP in Figure A detected by STEM; (**C**): A representative NP synthesized by pUC19-Met cells was chosen for compositional analysis; (**D**): Elements detected by STEM in NPs synthesized by pUC-Met cells.

**Figure 7:**
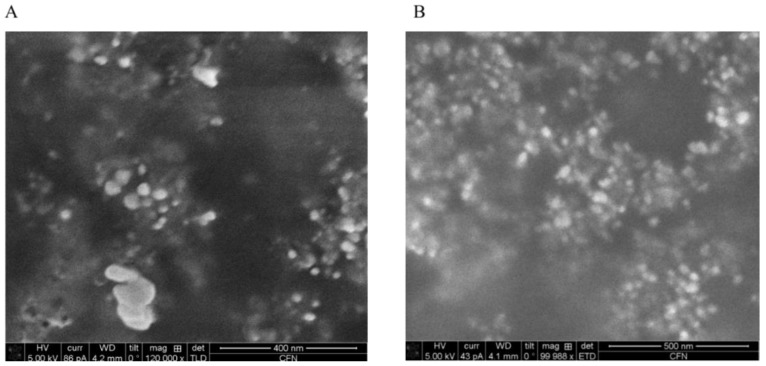
Dual-beam focus ion beam (FIB) SEM scanning of silver NPs. (**A**): NPs synthesized by pUC19-Vec cells under SEM; (**B**): NPs synthesized by pUC-Met cells.

**Figure 8:**
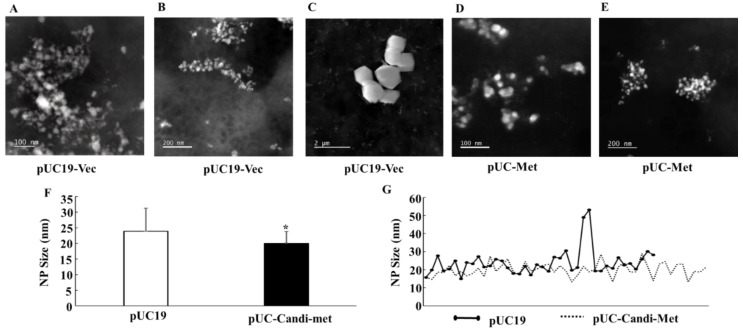
STEM analysis of NPs. (**A**,**B**): NPs formed by pUC19-Vec cells. (**C**). Large silver crystals synthesized by pUC19-Vec cells; (**D**,**E**): NPs formed by pUC-Met; (**F**): Bar graph showing the size of the NPs determined by STEM. The diameter or the longest side of isolated NPs was analyzed using Gatan Digital Micrograph Software; (**G**): The distribution of the size of the NPs synthesized by bacteria pUC19-Vec or pUC-Met. N = 40 NPs in pUC19-Vec; n = 49 NPs in pUC-Met. *: *p* < 0.001.

X-ray EDS compositional analysis identified that the major element in the particles was silver (Figure 6), confirming that the bacteria indeed synthesized silver NPs. Other elements found in the NPs included chloride, sulfur and copper (Figure 6). The detection of sulfur was particularly interesting, since it showed that the thiol groups of cysteine residues were in fact involved in reducing silver ions. Importantly, the analysis of the particle images also proved that all *E. coli* cells produced silver particles in the nano-size range (Figure 7 and Figure 8). The silver NP images in Figure 8 were selected for size analysis using Gatan Digital Micrograph Software. Figure 8 showed that the size of NPs produced by pUC19-Vec cells was slightly and significantly larger (Figure 8F,G. pUC19-Vec: 23.9 ± 7.3 nm. n = 40; pUC-Met: 20.0 ± 3.7 nm, n = 49. *p* < 0.05). Our results suggested that the NPs synthesized by pUC-Met cells were more homogeneous. Firstly, the size of the NPs formed by pUC-Met cells was more homogeneous, which was demonstrated by the difference in the standard deviation (SD) of the NP size. The SD of the size of NPs (not including the large crystals shown in Figure 8C) made by pUC19-Vec cells (SD = 7.3 nm) was much greater than that produced by pUC-Met cells (SD = 3.7 nm). Furthermore, the NPs produced by pUC-Met cells mostly had a round spherical shape (Figure 7B and Figure 8D,E); while the NPs synthesized by pUC19-Vec cells were more diverse (Figure 7A and Figure 8A–C). The heterogeneity of the NPs synthesized by the pUC19-Vec cells was further exemplified by the presence of some micro-size silver crystals with shapes of cube, rectangular and hexagonal prism (Figure 8C). Thus, the results of SEM and STEM examination demonstrated that the engineered cells synthesized more uniform silver NPs in both size and shape.

## 4. Discussion

Both wild type *E. coli* and engineered *E. coli* have been shown to be able to synthesize diverse NPs [9,10,11]; however, it is not clear whether the engineered *E. coli* cells perform better than non-engineered cells, and whether these cells have the potential to upgrade to large scale NP production. In this study, we explored the potential of application of *E. coli* DH5α cells transformed with *C. albicans* MT gene in the biosynthesis of more uniform silver NPs at lager scale. We used the *E. coli* DH5α cells transformed with pUC19 empty plasmid vector as a control, since the *C. albicans* MT gene was delivered into the cells by vector pUC19. Many studies have shown that MT is protective when cells are exposed to metal ions [6,17,18]. MT proteins have high binding-affinity for heavy metal ions and serve as important reducing agents under heavy metal stress; thus, MT proteins are critical for proper accumulation, distribution and detoxification of the metal ions [5,17,18]. Similarly, our results also showed that the *E. coli* cells expressing *C. albicans* MT (pUC-Met cells) tolerated CuSO_4_ challenge better than the pUC19-Vec cells. During the prolonged cultivation in the absence of metal ion stress, the OD_600nm_ of pUC-Met cells was higher than that of pUC19-Vec cells at 24, 48 and 72 h after the OD_600nm_ reached 0.6–0.8 (Figure 3A). Importantly, when the cells were subjected to copper ion stress for 17–19 h, the pUC-Met cells grew to a significantly higher OD_600nm_ than the pUC19-Vec cells (Figure 3B). In particular, assessment of cell viability confirmed that pUC-Met cells had a higher survival rate than pUC19-Vec cells after bacteria were exposed to heavy metal stress (Figure 3C). The better growth and higher survival of bacteria under heavy metal challenge suggested that the pUC-Met cells had increased tolerance to the metal ions, presumably due to the expression of *C. albicans* MT protein. *C. albicans* MT has a relatively high content of cysteine residues that can be used to reduce silver ions, bind and accumulates silver particles in the cells, thus, protect the cells from the toxicity of silver ions. We proposed that the higher tolerance to metal ions would subsequently promote the cells to make more NPs if they are used as biofactories for metal NP synthesis. Indeed, when the cells were grown in the presence of 1 mM AgNO_3_, the pUC-Met cells consistently produced more silver NPs at each examination time point (Figure 4A–E), and the final yield of NPs from 1liter culture by pUC-Met cells was greater than that of the pUC19-Vec cells (Figure 4E, 1 g/L vs. 0.74 g/L). The yield of a wet pellet of bacterial culture in the presence of 1 mM AgNO_3_ for 72 h was not compromised by increasing culture medium volume from 0.1 L to 1 L, suggesting that upgrade to large scale NP production is very promising, although more experiments are needed to confirm the conclusion. Thus, our results demonstrated that expression of *C. albicans* MT in *E. coli* promoted cell growth and survival and enhanced biosynthesis of silver NPs in these cells. 

Even if the mechanisms of bacterial synthesis of metallic NPs remain to be completely elucidated, it has been postulated that the presence of reducing groups that can donate electrons to metal ions, such as phenol, amide, thiols and other unsaturated bonds, of the organic matter in bacteria play an important role in reducing and binding metal ions [12,25]. To shed light into the mechanisms of bacterial NP synthesis, we analyzed the reducing functional groups using FTIR. FTIR identified compounds that probably contain phenol, amines, amide I and isothiocyanate functional groups in all cells (Figure 5). We hypothesized that the oxidization of C–OH group in organic molecules to C=O (amide I) group probably was involved in the reduction of Ag^+^ ions to Ag^0^. In addition, the amide I groups may cap and stabilize the silver NPs once the Ag^+^ ions were converted into Ag^0^. The possible amines (N–H) of biomolecules, such as proteins, may as well act as binding and stabilizing agents of silver NPs. Furthermore, it is well accepted that the O–H stretching in alcohols and phenolic compounds are used to reduce the Ag^+^ ions into Ag^0^ in the biological synthesis of metal NPs [22,23,24]. Therefore, the FTIR analysis suggested that the bacteria in our study probably used similar reducing agents to reduce Ag^+^ ions to Ag^0^ as reported [12,22,23,24,25]. Interestingly, a possible isothiocyanate (N=C=S) was observed in the bacteria synthesized silver NPs. Isothiocyanates are sulfur-containing antioxidants that have antimicrobial activities [26]. It is true that the strong reducing agent isothiocyanates can play a role in biosynthesis of silver NPs, it is yet to elucidate how and why the engineered bacteria produced these compounds that can kill themselves.

We expected that the FTIR could detect thiol groups, since the cysteine residues in the MTs contain thiol groups. However, FTIR could not catch the thiol groups associated with silver. We speculate that the thiol group, which usually is assigned to a weak peak around 2600 cm^−1^, might be buried in the stretches of the strong peaks adjacent to 2600 cm^−1^. To complement this drawback of FTIR, compositions of silver NPs were further assessed by STEM and SEM. Importantly, the compositional analysis confirmed that silver atoms were among the key elements in the NPs. Further, consistent with our prediction, sulfur atoms were indeed detected associated with these silver NPs, indicating that the thiol groups of proteins were involved in reducing silver ions and accumulating silver NPs (Figure 6). Therefore, our results not only confirmed that the NPs were indeed silver NPs, but also supported that thiol groups were involved in reducing and binding silver NPs. Since *C. albicans* MT has a higher level of cysteine residues that can generate a higher reducing power and metal-binding affinity in pUC-Met cells than the pUC-Met cells, we believe that the expression of *C. albicans* MT in *E*. *coli* cells allowed the cells to convert more silver ions, and faster into silver NPs, and therefore, protected the cells from silver ion stress, as well as increased the bacterial NP yield.

Another finding of the SEM compositional assessment was the detection of chloride and copper associated with silver NPs. It has been shown that chloride is one of the key elements that *E. coli* use to counter silver NP toxicity [27]. The identification of chloride associated with silver NPs suggested that the *E. coli* not only used thiol groups, but also chloride to reduce and accumulate silver under silver ion stress. It is not surprising that copper was also detected in the NPs, since *C. albicans* MT was originally discovered as a copper-binding protein, and its expression was induced by copper ions [17,18].

FTIR analysis, SEM and STEM examinations obtained similar results in the NPs synthesized by pUC19-Vec and pUC-Met cells, suggesting the two groups of cells probably used similar mechanisms to reduce silver ions and produce NPs. However, the expression of *C. albicans* MT protein in *E. coli* conferred the engineered recombinant cells higher tolerance and better survival, thus, greater capacity to synthesize silver NPs when exposed to silver ions.

One of the problems associated with biologically synthesized NPs has been morphological heterogeneity of the NPs [12,13,14]. A primary goal of this study was to investigate whether engineered *E. coli* cells can improve the quality of NPs. Both SEM and STEM demonstrated that the two groups of *E. coli* cells successfully produced silver particles in the nano-size range. The average size of NPs synthesized by pUC-Met cells is slightly, but significantly, smaller than the size of those produced by pUC19-Vec cells (Figure 8F,G). However, there was a greater variation in the sizes of the NPs synthesized by pUC19-Vec cells (SD: 3.7 nm in pUC-Met vs. 7.3 nm in pUC19-Vec). Note that calculation of this SD did not include the large crystals mentioned below). Surprisingly, some micro-size silver crystals were formed by these cells (Figure 8C). Overall, our data showed that NPs produced by pUC-Met cells not only had a more uniform size, but also appeared to have a more uniform shape. As shown in Figure 7B, Figure 8D,E, under SEM and STEM, it appeared that the NPs made by pUC-Met cells had a round shape, while the morphology of NPs from pUC19-Vec cells was more heterogeneous, which showed round, rectangular prism and hexagonal prism shapes (Figure 7A and Figure 8A,C). Thus, we demonstrated that both pUC19-Vec and pUC-Met bacterial groups were able to synthesize silver NPs. Nevertheless, the bacteria expressing *C. albicans* MT had a better control in the dimension and morphology of silver NPs and a higher yield of NPs. The mechanisms of how the pUC-Met recombinant bacteria synthesized NPs with a more uniform size and shape remains to be elucidated. We speculate that more thiol groups in pUC-Met cells have a higher reducing power and a greater binding capacity for silver ions, which probably allows the cells to produce NP faster; meantime, more thiol groups can provide a larger silver binding capacity, which is more potent to stabilize the silver NPs once the silver ions are reduced. Faster reducing reactions along with a higher capacity to stabilize the NPs probably allowed the cells to synthesize NPs with a smaller size and higher uniformity. 

## 5. Conclusions

In summary, we revealed that the expression of *C. albicans* MT in *E. coli* cells allowed these cells to biologically synthesize more silver NPs at a higher yield. An important finding is that these engineered recombinant *E. coli* cells appeared to have better control in the size and shape of the NPs. Moreover, the procedures used to grow, propagate the engineered bacteria, as well as the methods used to produce silver NPs, in our study, are quite simple and easy to set up in any microbiology laboratories. Expansion of this engineered *E. coli* as a platform for the biosynthesis of other metal NPs, optimization of bacterial growth conditions to upgrade the method for large scale production of uniform metal NPs, as well as an exploration of the potential of in vitro NP synthesis by these engineered cells will be among our goals in the future. The results of this study provided preliminary information for genetically modifying bacteria as promising biofactories for large-scale production of size- and shape-controlled NPs for their applications in nanotechnology.
